# Small RNA F6 Provides *Mycobacterium smegmatis* Entry into Dormancy

**DOI:** 10.3390/ijms222111536

**Published:** 2021-10-26

**Authors:** Artem Grigorov, Oksana Bychenko, Elena G. Salina, Yulia Skvortsova, Arina Mazurova, Timofey Skvortsov, Arseny Kaprelyants, Tatyana Azhikina

**Affiliations:** 1 Shemyakin-Ovchinnikov Institute of Bioorganic Chemistry, Russian Academy of Sciences, 117997 Moscow, Russia; artgrigorov@gmail.com (A.G.); oksana.belonovich@gmail.com (O.B.); ju.skvortsova@gmail.com (Y.S.); mazarina@yandex.ru (A.M.); 2 Research Center of Biotechnology, Bach Institute of Biochemistry, 119071 Moscow, Russia; elenasalina@yandex.ru (E.G.S.); arseny@inbi.ras.ru (A.K.); 3 School of Pharmacy, Queen’s University Belfast, Belfast BT9 7BL, UK; t.skvortsov@qub.ac.uk

**Keywords:** *Mycobacterium smegmatis*, small non-coding RNA, F6, resuscitation promoting factor RpfE2, dormancy, non-culturability, adaptation to stresses

## Abstract

Regulatory small non-coding RNAs play a significant role in bacterial adaptation to changing environmental conditions. Various stresses such as hypoxia and nutrient starvation cause a reduction in the metabolic activity of *Mycobacterium smegmatis*, leading to entry into dormancy. We investigated the functional role of F6, a small RNA of *M. smegmatis*, and constructed an F6 deletion strain of *M. smegmatis*. Using the RNA-seq approach, we demonstrated that gene expression changes that accompany F6 deletion contributed to bacterial resistance against oxidative stress. We also found that F6 directly interacted with 5′-UTR of *MSMEG_4640* mRNA encoding RpfE2, a resuscitation-promoting factor, which led to the downregulation of RpfE2 expression. The F6 deletion strain was characterized by the reduced ability to enter into dormancy (non-culturability) in the potassium deficiency model compared to the wild-type strain, indicating that F6 significantly contributes to bacterial adaptation to non-optimal growth conditions.

## 1. Introduction

Bacteria are exposed to various stresses during their lifetime and, in order to adapt and survive, they have to rapidly modify their gene expression. To achieve this, bacteria employ small regulatory RNAs (sRNAs), which post-transcriptionally regulate bacterial gene expression and provide rapid responses to changes in environmental conditions such as nutrient deprivation, stresses, or host responses [1,2,3]. Recent evidence shows that sRNAs act as signal transducers of environmental cues, participate in regulatory networks, and precisely coordinate gene expression in many adaptation processes by controlling mRNA transcription, translation, and stability [2]. These diverse functions are performed through various mechanisms, including RNA conformational changes, interaction with proteins, and complementary interactions with other RNA or DNA molecules.

In mycobacteria, sRNAs have been identified relatively recently [4], which can be attributed to mycobacterial physiology, as well as the absence of Hfq or other chaperones. Recent technical advances, including high throughput sequencing and computer algorithms, have enabled the identification of dozens of sRNAs in mycobacteria [5,6,7,8,9]. To date, sRNAs have been detected and mapped in several mycobacterial species, including *Mycobacterium tuberculosis*, *M. bovis*, *M. smegmatis*, *M. marinum*, and *M. avium* [6], and a significant number of them has been confirmed experimentally [10,11,12]. However, the regulatory mechanism is established for only a few mycobacteria (reviewed in [7,8]).

SRNA F6 (ncRv10243, MTS194, MTB000051) was discovered in *M. tuberculosis* by sequencing of the low molecular mass RNA fraction and confirmed by Northern blotting [4]. F6 was found to be conserved in many species of the genus *Mycobacterium*, both pathogenic slow-growing and non-pathogenic fast-growing. In *M. tuberculosis*, the upregulation of F6 expression was detected under oxidative stress, hypoxia, low pH conditions, and macrophage infection, but the strongest upregulation was observed with nutrient deficiency [4,13]. The overexpression of F6 suppressed the growth of *M. tuberculosis* cells; however, neither overexpression nor deletion of the F6 gene affected the growth of *M. smegmatis* cells [4]. The deletion of F6 prevented the transition of *M. tuberculosis* from the dormant state in the Wayne hypoxia model [13]. The function of F6 under starvation has been elucidated in a recent study, which shows that F6 activates the expression of *Rv0440* (groEL2) and *Rv3418c* (groES).

In this study, we examined the functional role of F6 in *M. smegmatis*, a free-living non-pathogenic bacterium. *M. smegmatis* has 79% nucleotide sequence identity with *M. tuberculosis* and is similar to it in terms of cell wall composition and metabolism [14], but differs significantly in lifestyle. By using a combination of bioinformatic target prediction and RNA-seq of the F6 knockout (ΔF6) strain, we found that the only candidate target was *MSMEG_4640*. F6 directly interacted with the 5′-UTR of *MSMEG_4640* mRNA, decreasing the amount of the encoded protein, RpfE2, which belongs to the resuscitation-promoting factor (Rpf) family. Proteins of the Rpf family are hydrolytic enzymes that, similar to lysozyme and lytic transglycosylases, can cleave 1→4 glycosidic bonds between N-acetylglucosamine and N-acetyl (glycolyl) muramic acid of bacterial peptidoglycan [15]. Being non-essential for the active growth of *M. tuberculosis*, Rpfs determine the transition of dormant forms to the active state both in vitro [16] and in vivo [17]. Here, we showed that in the in vitro model of *M. smegmatis* dormancy and “non-culturability” under potassium-limiting conditions [18], the ΔF6 strain, in contrast to the wild-type (wt) strain, demonstrated the ability to remain culturable under stress. These results indicate the involvement of RpfE2 and F6 sRNA in the transition of *M. smegmatis* to dormancy under non-optimal growth conditions, suggesting distinct functional roles of F6 in *M. smegmatis* and *M. tuberculosis*.

## 2. Results

### 2.1. Deletion of F6 Does Not Affect Bacterial Growth

**Figure 1:**
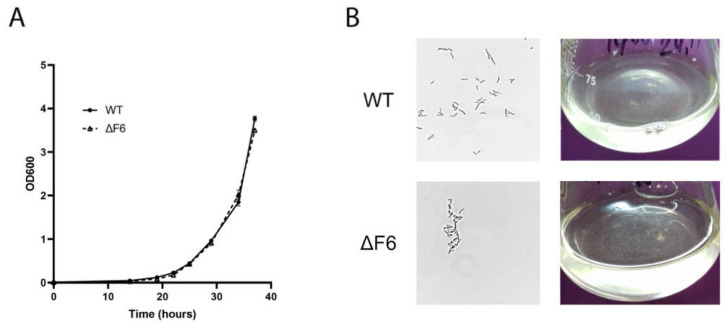
(**A**) Growth curves of *M. smegmatis* wt and ΔF6 in Sauton’s medium supplemented with 0.05% (*v*/*v*) Tween 80. The data are presented as the mean ± SD of three independent experiments. (**B**) Light microscopy image of wt and ΔF6 *M. smegmatis* cells in the early-log phase; magnification ×1250 (left panels) and the image of wt and ΔF6 cultures growing in liquid media (right panels).

To obtain the unmarked F6 knockout strain (ΔF6) of *M. smegmatis*, we employed the p2NIL/pGOAL allele replacement procedure widely used in genetic engineering of mycobacteria [19,20]. The scheme is shown in Supplementary Figure S1. The homologous regions flanking the F6 gene (left, 416,281–417,720 bp and right, 417,885–419,360 bp according to the *M. smegmatis* genome [GenBank accession number NC_008596.1]) were amplified and cloned into the p2NIL vector. The two-step strategy was based on kanamycin, hygromycin, and X-gal selection at the plasmid integration stage with subsequent sucrose and X-gal selection to produce double crossovers. The resulting clones were checked by PCR for the absence of the F6 gene (Supplementary Figure S1). Sanger sequencing of these PCR products confirmed F6 deletion in the region of 417,720–417,885 bp. To determine whether F6 deletion affected the *M. smegmatis* phenotype, the growth of the wt and ΔF6 strains was compared in Sauton’s medium supplemented with ADC and Tween-80 (Figure 1A). The results indicated that there were no obvious differences in the growth of the two strains. Light microscopy analysis revealed that in the early logarithmic phase, ΔF6 cells were prone to aggregation (Figure 1B), which may indicate that the deletion of F6 altered the cell wall composition of *M. smegmatis*. The aggregation disappeared with the growth of cell cultures.

### 2.2. F6 Deletion Affects Gene Expression in M. smegmatis

**Figure 2:**
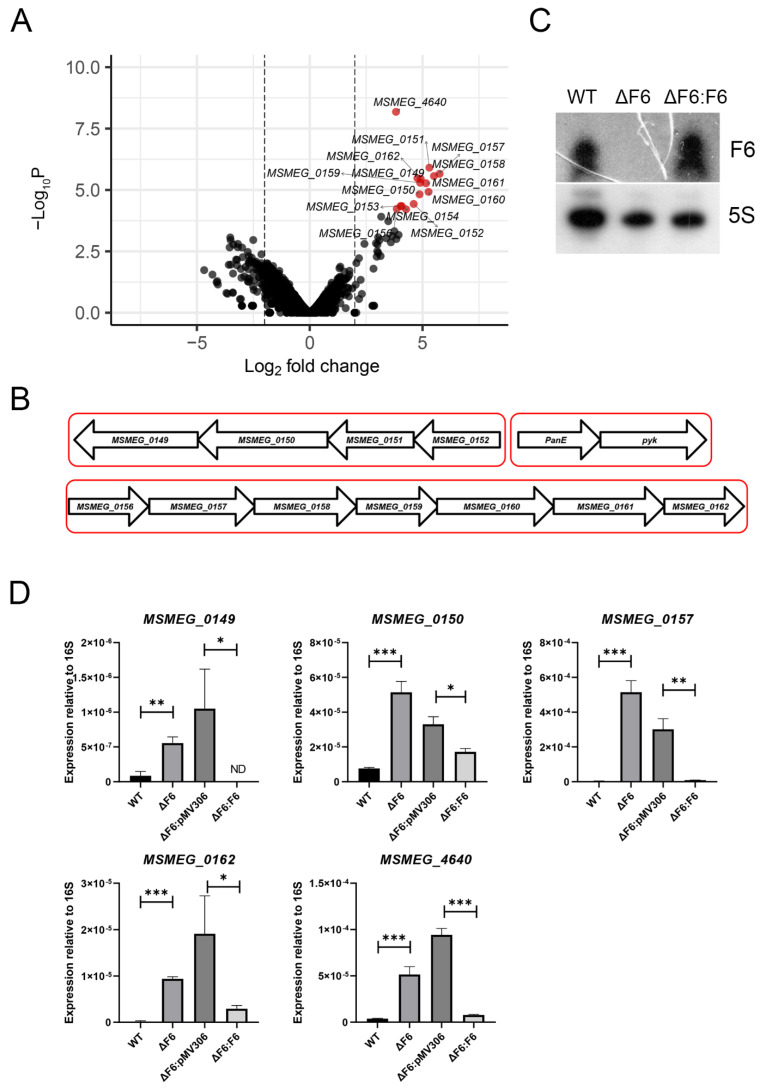
RNA-seq of the wt and ΔF6 strains. (**A**) Volcano plot of differentially expressed genes (DEGs) constructed using Enhanced Volcano R package [24]. Fold changes of gene expression were plotted. Significant DEGs were identified by >four-fold change (log2 FC > 2) and <0.05 FDR, and are shown in red. (**B**) Schematic representation of the gene clusters upregulated in the ΔF6 strain. (**C**) Northern blotting analysis of F6 transcription in the wt, ΔF6, and F6 complemented (ΔF6:F6) strains. (**D**) Validation of DEGs by qRT-PCR. mRNA expression was determined in the wt, ΔF6, ΔF6:pMV306, and ΔF6:F6 cultures in the mid-log phase and normalized to that of 16S rRNA. * *p* < 0.05, ** *p* < 0.01, and *** *p* < 0.001. The data are presented as the mean and SD of three biological replicates for each strain.

To examine the differences in gene expression caused by F6 deletion, we performed transcriptome sequencing of the wt and ΔF6 strains in the mid-log growth phase. Earlier, it had been demonstrated that F6 is highly expressed in the logarithmic growth phase both in *M. tuberculosis* [4] and *M. smegmatis* (our unpublished data). The sequencing reads were mapped against the reference genome (GenBank accession number NC_008596) and the details of mapping are given in Supplementary Table S1. Overall, 15 genes were found to be differentially expressed (FC ≥ 4, *p* < 0.05), all of which were upregulated in the ΔF6 strain (Figure 2A). The list of differentially expressed genes (DEGs) is given in Table 1.

Two large gene clusters could be distinguished (Figure 2B). The first cluster (*MSMEG_156-162*) comprised seven genes, which were upregulated by more than 30-fold in the ΔF6 strain and the majority of which (*MSMEG_0158-MSMEG_0162*) encoded enzymes of formate metabolism. *MSMEG_0157* is responsible for the utilization of oxalate, a toxic substance incorporated in the energy metabolism of some bacteria. In *M. tuberculosis*, the *MSMEG_0157* homologue is essential for bacterial growth [21].

Another gene in this cluster was *MSMEG_0156*, encoding a transcription factor of the LysR family. The homologue of this gene in *M. tuberculosis* is *OxyS*, involved in the regulation of responses to oxidative stress by decreasing the expression of *katG* and *ahpC* genes coding for catalase-peroxidase and alkyl hydroperoxide reductase, respectively; therefore, the upregulation of this transcription factor makes bacteria more sensitive to peroxides [22].

The second upregulated cluster consisted of *MSMEG_0149-MSMEG_0152* and included proton-translocating NAD(P)(+) transhydrogenase PntAB (*MSMEG_0151*), which functions as a proton pump across the membrane and plays a role in the adaptation of bacteria to acid/oxidative stress [23].

To confirm the RNA-seq results, we performed quantitative (q)RT-PCR of several DEGs on RNA templates isolated from the wt, ΔF6, and complemented (ΔF6:F6) strains in the mid-log phase. The complemented strain was constructed by transforming the ΔF6 strain with the integrative expression plasmid F6_pMV306, where F6 transcription was driven by the rrnB promoter of *M. smegmatis*. The absence of F6 transcription was confirmed by Northern blotting (Figure 2C), which detected no F6 signal in the ΔF6 strain. The results demonstrated that *MSMEG_0149*, *MSMEG*_*0150*, *MSMEG*_*0157*, *MSMEG_0162,* and *MSMEG*_*4640* were significantly upregulated after F6 deletion in the ΔF6 strain (Figure 2D).

### 2.3. F6 Directly Targets 5′-UTR of MSMEG_4640 mRNA

**Figure 3:**
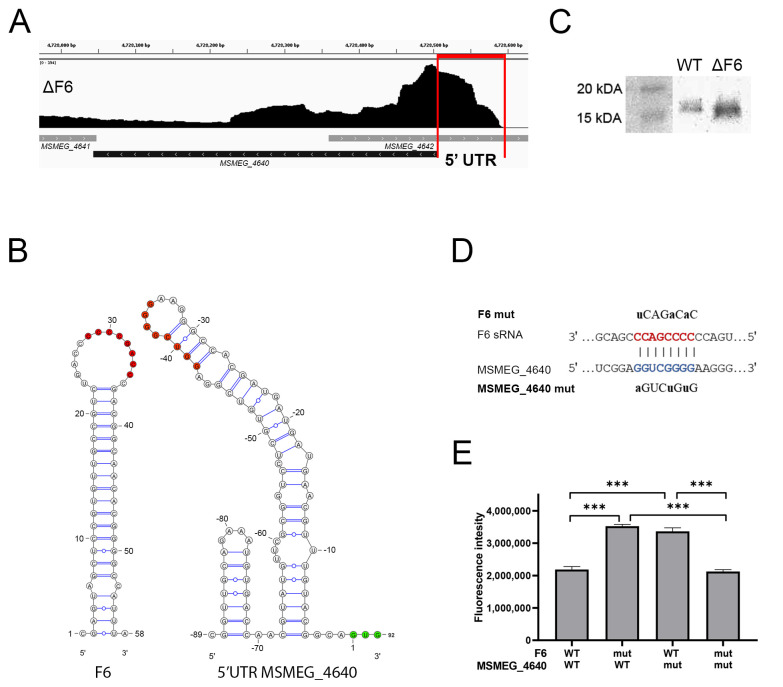
F6 of *M. smegmatis* directly interacts with the 5′-UTR of *MSMEG_4640.* (**A**) Inhibition of RpfE2 protein expression by F6. The wt and ΔF6 cultures were analyzed for RpfE2 expression by Western blotting using antibodies against the Rpf conserved domain. The RpfE2 molecular mass is 15.1 kDa according to Mycobrowser data (https://mycobrowser.epfl.ch/genes/MSMEG_4640, accessed on 28 June 2021). (**B**) The coverage track of the *MSMEG_4640* locus in Integrative Genomics Viewer [25]. RNA-seq data of the ΔF6 strain in the mid-log growth phase are shown. The 5′-UTR is marked by vertical red lines. (**C**) Secondary structures of F6 and the 5′-UTR of *MSMEG_4640*. The interacted nucleotides are shown as red dots; green dots mark the start codon. (**D**) Schematic representation of the interaction between F6 and its target *MSMEG_4640*. The F6 seed region is in red and the complementary 5′-UTR region is in blue. The introduced mutations are shown above and below. (**E**) The reporter assay illustrating the direct regulation of *MSMEG_4640* by F6. The 5′-UTR of *MSMEG_4640* was fused to the *GFP* gene and reciprocal mutations were introduced in the putative interaction sites on F6 and *MSMEG_4640*-GFP. GFP translation was estimated by fluorescence. The data are presented as the mean ± SD of three biological replicates for each strain; *** *p* < 0.001.

To identify mRNA targeted by F6, we selected potential targets using the CopraRNA prognostic software (http://rna.informatik.uni-freiburg.de, accessed on 20 May 2019), and compared the list of targets with that of DEGs according to RNA-seq. The only gene present in both lists was *MSMEG_4640*. F6 deletion caused the upregulation of *MSMEG_4640* at both the mRNA and protein levels, which was confirmed by qRT-PCR (Figure 2D) and Western blotting (Figure 3A) of wt and ΔF6 cells.

The RNA-seq profiles revealed that *MSMEG_4640* mRNA contained a ~90 nt-long 5′-UTR (Figure 3B), which had a secondary structure with a loop from −30 to −38 nt upstream of the start codon; this 8-nt loop was complementary to the F6 loop, thus enabling the formation of an intermolecular duplex (Figure 3C).

To examine whether F6 directly interacted with the 5′-UTR of *MSMEG_4640* mRNA, we performed an expression reporter assay with the *GFP* gene, which was fused to the 5’-UTR fragment. The integrative plasmid carrying this reporter was transfected into the wt and ΔF6 strains. Then, we introduced point mutations in the F6 seed region and the putative binding site in the 5′-UTR, and evaluated their effects on GFP expression by measuring fluorescence. In cases of complete complementarity (‘wt vs. wt’ and ‘mut vs. mut’; Figure 3D,E), GFP expression was lower than in partly complementary duplexes (‘wt vs. mut’ and ‘mut vs. wt’), indicating that mutations destroyed the duplex and abrogated the regulation of target gene expression. These results demonstrated that F6 regulated *MSMEG_4640* expression through direct interaction between the 8-nt F6 seed sequence and the perfectly complementary region in the 5′-UTR of the *MSMEG_4640* mRNA.

### 2.4. F6 Expression Abrogates M. smegmatis Entry into Dormancy

**Figure 4:**
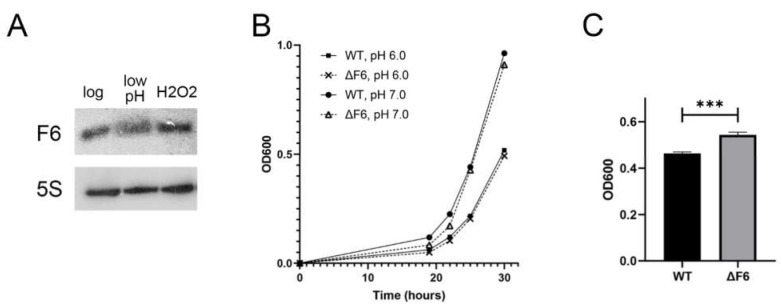
Effects of oxidative and acidic stresses on the *M. smegmatis* ΔF6 and wt strains. (**A**) F6 transcription in the mid-log growth phase and under acidic (low pH) and oxidative (H_2_O_2_) stresses was analyzed by Northern blotting. (**B**) Growth at neutral (pH 7) and acidic (pH 6) conditions. (**C**) Growth under oxidative stress (0.5 mM H_2_O_2_) at eight hours after H_2_O_2_ addition. The data are presented as the mean ± SD of three independent experiments; *** *p* < 0.001.

According to a previous study [4], F6 expression in *M. tuberculosis* increases approximately two-fold by acid and oxidative stresses. To compare F6 expression in *M. tuberculosis* and *M. smegmatis*, we evaluated F6 expression in the wt strain under low pH and oxidative stress by Northern blotting. The results revealed that F6 expression was only slightly reduced by low pH and was not changed after H_2_O_2_ treatment (Figure 4A).

Then, we compared the growth of the wt and ΔF6 strains under oxidative and acid stresses. There were no significant differences between the wt and ΔF6 strains after acid stress (Figure 4B); however, the ΔF6 strain showed faster growth than the wt strain after H_2_O_2_ treatment (Figure 4C), which could be attributed to the induction of the OxyR regulon controlling the oxidative stress response in mycobacteria [22].

**Figure 5:**
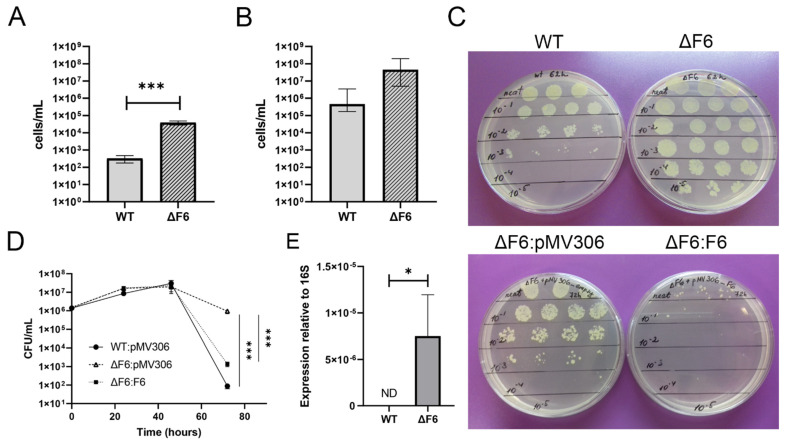
F6 regulates the transition of *M. smegmatis* to dormancy under growth-limiting conditions. (**A**) Colony-forming unit (CFU) concentration for *M. smegmatis* wt and ΔF6 strains growing in potassium-limiting conditions at the point of minimal culturability (65 h). (**B**) Reactivation of dormant ‘non-culturable’ *M. smegmatis* cells in the wt and ΔF6 strains co-cultured with *M. luteus* at the point of minimal culturability (65 h) in standard Sauton’s medium (most probable numbers, MPN). (**C**) Culturability of *M. smegmatis* wt and ΔF6 strains under potassium-limiting conditions. (**D**) Growth of *M. smegmatis* wt, ΔF6:pMV306, and ΔF6:F6 strains in potassium-deficient medium. (**E**) qRT-PCR analysis of *MSMEG_4640* expression in wt and ΔF6 cultures growing under potassium limiting conditions (45 h). The data are presented as the mean ± SD of four (**A**,**B**,**D**) or three (**E**) biological replicates for each strain; * *p* < 0.05 and *** *p* < 0.001.

Thus, our results demonstrated that F6 directly targeted the 5′-UTR of *MSMEG_4640* and regulated its expression. To check whether F6 affects expression and biological function of RpfE2, we tested the ability of the ΔF6 *M. smegmatis* strain to transit to the dormant non-culturable state [18] and to resuscitate from dormancy. Because *rpf* genes in mycobacteria are involved in these processes [16,26], possible phenotypic changes might be noticed in the specific dormancy-resuscitation system rather than during active bacterial growth in vitro. Rpf-mediated resuscitation of dormant ‘non-culturable’ bacteria of both the wt and ΔF6 strains was performed by co-cultivation with exponentially growing *Micrococcus Luteus*, which secretes Rpfs, according to the procedure described earlier [18]. The strains demonstrated similar abilities to transit from the non-culturable to the culturable state (Figure 5A,B). However, a phenotypic difference between the strains was found in the process of entering dormancy as the ΔF6 strain demonstrated significantly higher culturability under stress than the wt strain (Figure 5C,D); furthermore, analysis of *MSMEG_4640* expression revealed transcripts only in the ΔF6 strain (Figure 5E).

F6 complementation was found to partially restore the ability of the mutant to form dormant ‘non-culturable’ cells under potassium-limiting conditions (Figure 5C,D), indicating the involvement of RpfE2 and F6 sRNA in maintaining *M. smegmatis* culturability in stressful conditions and in ability to transit to dormancy.

## 3. Discussion

Despite differences in many aspects such as lifestyle and growth rate, *M. smegmatis* and *M. tuberculosis* share a sufficient number of highly homologous sRNAs. A combined computational and experimental approach has identified sRNAs from *M. tuberculosis* and *M. smegmatis* and revealed that the expression of many sRNAs is conserved across the mycobacterial species [10].

In this study, we investigated F6, an *M. smegmatis* sRNA which is highly conserved among mycobacterial species, including *M. tuberculosis*, where F6 has been characterized [4,13]. The Arnvig’s group has demonstrated that in *M. tuberculosis,* F6 is upregulated during starvation through the activity of transcription factor SigF, and in turn activates the synthesis of chaperonins GroES and GroEL2, promoting *M. tuberculosis* survival in granulomas [13].

Comparison of the F6 promoter regions in *M. tuberculosis* and *M. smegmatis* shows that the SigF binding site (−35 nt) characteristic for *M. tuberculosis* disappears in the *M. smegmatis* genome, where it is changed for the SigD consensus GTAACG [27]. In mycobacteria, SigD expression is upregulated under starvation and decreases during hypoxia [28]. The F6 regulation in mycobacteria should be further investigated, but in *M. smegmatis* this sRNA is not controlled by SigF. 

In search for potential mRNA targets of F6 in *M. smegmatis*, we constructed an unmarked F6 deletion strain and used RNA-seq to determine DEGs in the F6 knockout strain compared to the wt strain. Among the 15 DEGs, which were all upregulated in the ΔF6 mutant, the overwhelming majority (*MSMEG_0157-MSMEG_0162*) are under control of the oxidative stress response regulator OxyS (*MSMEG_0156*) [22]. The ΔF6 strain grew faster than the wt strain under oxidative stress (Figure 4B), which was possibly due to the upregulation of these genes. However, we have not yet determined any direct connection between this phenomenon and F6 deletion.

By using the combination of RNA-seq data and bioinformatic predictions, we found that F6 targeted the 5′-UTR of *MSMEG_4640* mRNA and that the direct interaction between the F6 seed with the target led to the downregulation of *MSMEG_4640*-encoded protein RpfE2. The search for the phenotype associated with the activity of F6 sRNA revealed that under non-optimal conditions induced by potassium deficiency, the mutant strain continued to grow, whereas the wt strain entered dormancy and the non-culturable state.

RpfE2 encoded by *MSMEG_4640* is a secreted protein belonging to the family of resuscitation-promoting factors (Rpfs) that act as growth stimulators through their lysozyme-like activity towards peptidoglycan in the bacterial cell wall [29]. The secreted Rpf initially found in *Micrococcus luteus* has been shown to initiate the reactivation of dormant cells and stimulate the replication of growing cells [30,31], including G+C-rich bacilli, particularly of the genus *Mycobacterium* [30]. Rpf-like secreted proteins characterized by the presence of a conservative domain of 75 amino acids have been found in some other *Actinobacteria* species. *M. smegmatis* has four and *M. tuberculosis*—five *rpf* genes.

Recombinant Rpfs have been shown to effectively stimulate the reactivation of dormant ‘non-culturable’ *M. tuberculosis* [32,33] and *M. smegmatis* [18] cells. In contrast to *Micrococcus luteus*, KO mutants for *rpf* genes in *M. tuberculosis* are viable both in vitro and in vivo. A mutation in one of the five genes encoding Rpfs in *M. tuberculosis* do not stop cell growth or affect the reactivation process, indicating certain redundancy in the activity of Rpfs and their mutual compensatory effects; however, such mutations can cause changes in cell morphology [16,34].

To find the functional meaning of the F6-*rpfE2* interaction, we used the *M. smegmatis* dormancy model when mycobacteria are grown in nutrient-inappropriate potassium-deficient medium resulting in a stable non-culturable state [18]. It was demonstrated that resuscitation of these dormant *M. smegmatis* has Rpf-mediated nature [18].

Rpf proteins in *M. smegmatis* have not been thoroughly studied yet. The relationship between biofilm formation and Rpfs in *M. smegmatis* has been recently demonstrated by Ealand et al. [35]; they observed that simultaneous deletion of *rpf* genes hampered the development of biofilms and reduced drug tolerance, and that these effects were accompanied by a decrease in muropeptide production and altered peptidoglycan cross-linking. Recently, the same group has examined the role of *M. tuberculosis* Rpfs in reactivation processes by expressing them in *M. smegmatis* [36]. Their results indicate that the growth stimulatory effect observed with the culture filtrate is most likely the result of a combination of Rpfs with other factors [36]. In our study, the changes in RpfE2 expression had a dramatic effect on *M. smegmatis* entering into the ‘non-culturable’ state but did not affect its reactivation from dormancy.

Despite many attempts to clarify the processes of mycobacteria entry into and reactivation from the dormant state, they are still poorly understood. Our results indicate that the ability of *M. smegmatis* to switch to the dormant ‘non-culturable’ state in order to survive in stressful surroundings is regulated by F6 sRNA, which directly interacts with *MSMEG_4640* mRNA, inhibiting the expression of the encoded resuscitation factor RpfE2. Thus, *M. smegmatis* F6 sRNA may contribute to bacterial tolerance to and persistence in stressful environmental conditions.

## 4. Materials and Methods

### 4.1. Bacterial Strains, Media, and Growth Conditions

*M. smegmatis* mc^2^155 cells from the bacterial collection of the Bach Institute of Biochemistry (Research Center of Biotechnology of the Russian Academy of Sciences, Moscow, Russia) and mutant *M. smegmatis* strains were pre-cultured from frozen stock in Nutrient Broth rich medium (Himedia, India) supplemented with 0.05% (*v*/*v*) Tween 80 for 30 h at 37 °C on an orbital shaker (200 rpm), and then regrown in Sauton’s medium [37] supplemented with 0.05% Tween 80.

For stress survival experiments, *M. smegmatis* mc^2^155 were grown to the early logarithmic phase (OD_600_ = 0.3) in Sauton’s medium supplemented with 0.05% Tween 80. To simulate acidic or oxidative stresses, cultures were incubated at 37 °C on an orbital shaker (200 rpm) with HCl (5 µM) for 2 h or with H_2_O_2_ (5 µM) for 8 h, respectively. Control cultures were grown in parallel with the stressed cultures.

For cloning procedures, *Escherichia coli* DH5α was grown in Luria Bertani (LB) broth and LB-agar. 

Oligonucleotides, plasmids and strains used are listed in Supplementary Table S2.

### 4.2. Construction of F6 Deletion (ΔF6) and Complementation (ΔF6::F6) Strains

The allelic replacement technique was used to generate an *M. smegmatis* knockout mutant as described previously [20]. Briefly, the right (1476 bp) and left (1440 bp) regions flanking the deletion site were amplified from genomic DNA using primers RHA_F6_for/RHA_F6_rev and LHA-F6_for/LHA-F6_rev and the obtained PCR products were inserted into the p2NIL delivery plasmid at *Bam*HI and *Hind*III restriction sites, respectively. The *sacB* and *lacZ* selection genes from the pGOAL19 vector were inserted into p2NIL at the *Pac*I restriction site to yield the pF6_new_Knockout suicide plasmid (Supplementary Figure S1), which was used to transform electrocompetent *M. smegmatis* mc^2^155 cells by standard methodology. During the first homologous recombination, clones were selected on a medium containing kanamycin (20 μg/mL), hygromycin (50 μg/mL), and 5-bromo-4-chloro-3-indolyl-β-D-galactopyranoside (X-gal, 0.4%). To obtain a double crossover, the resulting colonies were scattered into cups containing sucrose (2% *w*/*v*) and X-gal, and white colonies were screened for resistance to hygromycin and kanamycin.

Selected colonies were checked for deletion by PCR with primers F6-KO-check_for and F6-KO-check_rev, and the obtained amplicons were sequenced by the Sanger procedure (Evrogen, Moscow, Russia).

To complement the ΔF6 strain, electrocompetent cells were transformed with the pMV306_F6 integrating plasmid containing the F6 gene under the rrnB mycobacterial promoter.

### 4.3. RNA Isolation

Bacterial cultures were grown up to the logarithmic phase (OD_600_ = 1.0) and centrifuged (4 °C, 4000 rpm, 15 min). The pellets were washed twice with fresh medium, rapidly cooled on ice, and centrifuged again. Total RNA was isolated by phenol-chloroform extraction and cell disruption using Bead Beater (BioSpec Products, Bartlesville, OK, USA) as described previously [38] and treated with Turbo DNase (Life Technologies, Carlsbad, CA, USA) to remove traces of genomic DNA. RNA quantity and purity were determined spectrophotometrically and its integrity was assessed by electrophoresis in 1% agarose gels.

### 4.4. cDNA Synthesis and qRT-PCR

CDNA was synthesized from 1 mg total RNA using random hexanucleotides and SuperScript III reverse transcriptase (Life Technologies, USA) according to the manufacturer’s protocol, and used as a template in qRT-PCR performed with qPCRmix-HS SYBR (Evrogen) in a LightCycler 480 Real-Time PCR system (Roche, Switzerland) at the following cycling conditions: 95 °C for 20 s, 61 °C for 20 s, and 72 °C for 30 s, repeated 40 times. Three biological and nine technical replicates were used to ensure reproducibility, and the results were analyzed by LinRegPCR v 2014.6. The data were normalized against 16S rRNA to correct for sample-to-sample variation and the relative expression ratios were determined as described earlier [39].

### 4.5. Northern Blotting

To detect F6, 2 μg of total RNA isolated from exponential bacterial cultures was separated on 10% denaturing polyacrylamide gels in 1× TBE buffer and transferred to Hybond N Membranes (Amersham, UK). The membranes were hybridized overnight at 42 °C in ULTRAhyb-Oligo hybridization buffer (Life Technologies) with oligonucleotides F6_NB and 5S_NB, which were 5′-end-radiolabeled (15 pmoles) using 10 μCi of [γ32P]-ATP and T4 polynucleotide kinase (Fermentas, Lithuania). After hybridization, the membranes were washed three times in 1× saline-sodium citrate buffer with 0.1% SDS and exposed to X-ray films Retina (Carestream Health, Rochester, NY, USA) to detect radioactivity.

### 4.6. Western Blotting 

Bacterial cells were lysed using Bead Beater (BioSpec Products) and heated for 5 min at 95 °C in 2× sodium dodecyl sulfate (SDS) sample buffer (100 mM of Tris-HCl, pH 6.8, 4% SDS, 0.2% Bromophenol Blue, 20% glycerol, and 200 mM of DTT). Protein concentration was measured by the Bradford assay. Equal amounts of total protein (5 μg) were resolved by SDS-PAGE in a 12% gel and transferred onto Hybond-P membranes (GE Healthcare, Little Chalfont, UK), which were blocked with 5% *w/v* nonfat dry milk (Bio-Rad, Hercules, CA, USA) and incubated with primary antibodies against the conservative Rpf domain [31] and then with secondary anti-rabbit horseradish peroxidase (HRP)-conjugated IgG (Cell Signaling Technology, Beverly, MA, USA). Specific signals were visualized using a Clarity Western ECL (Bio-Rad) in a Bio-Rad ChemiDoc Touch imager station.

### 4.7. Libraries for RNA-Seq and Data Analyses

RNA samples were depleted of rRNA using the Ribo-Zero rRNA Removal Kit (Bacteria) (Epicentre, Madison, WI, USA) and used to generate sequencing libraries with the NEBNext Ultra II Directional RNA Library Prep Kit (NEB, USA) according to the manufacturers’ protocol. Sequencing was performed in triplicate using the Illumina HiSeq2500 as the pair-ended 100 nt reads. After quality control evaluation, the reads were mapped on the reference *M. smegmatis* genome (NC_008596.1, http://www.ncbi.nlm.nih.gov/, accessed on 18 May 2019) by Bowtie2 [40]; the alignment was performed with the “-local” and “-dovetail” options. Calculation of the mapped fragments for all genes was performed using the featureCounts program from the package Subread [41]. Only unambiguously mapped non-chimeric fragments were used in the subsequent analysis.

DEGs were identified by the edgeR software package [42]. The genes were considered to be differentially expressed if the FDR value was ≤0.05 and the expression change module (fold change, FC) was ≥4.

### 4.8. RNA-Seq and Visualization of the RNA Secondary Structure

Visualization of the depth of RNA sequencing coverage was carried out in the IGV genomic browser [25] using the deepTools tool suite [43]. The volcano plot of DEGs was constructed using the EnhancedVolcano R package [24].

Secondary structures of F6 and *MSMEG_4640* 5′-UTR were determined by the RNAfold web server [44] and visualized using VARNA applet [45].

### 4.9. GFP Fluorescence Assay

The following *M. smegmatis* strains were used for analysis: Msm∆F6__F6_MSMEG4640_5’utr_::GFP (as ‘wt vs. wt’), Msm∆F6__F6mut_MSMEG_4640_5’utr mut_:: GFP (as ‘mut vs. mut’), Msm_ MSMEG4640_5’utrmut_::GFP (as ‘wt vs. mut’), and Msm∆F6__F6mut _MSMEG4640_5’utr_::GFP (as ‘mut vs. wt’). Strains’ descriptions are given in Supplementary Table S2. 

To measure fluorescence, the strains were grown to the logarithmic phase (OD_600_ = 0.8) in LB broth supplemented with 0.05% Tween 80 and kanamycin (25 μg/mL). Cells were pelleted by centrifugation, washed twice, resuspended in 1× PBS buffer, and lysed using Bead Beater (BioSpec Products) as described previously [38]. The lysate was centrifuged (4 °C, 4000 rpm, 5 min) and the supernatant transferred to 96-well plates (200 μL per well). GFP fluorescence was measured on a Tecan ™ GENios^®^ Microplate Reader fluorometer (Tecan, Salzburg, Austria) with excitation and emission wavelengths of 488 and 510 nm, respectively. The results were expressed as the mean ± SD, calculated based on three biological replicates.

### 4.10. Dormant M. smegmatis Cells

Cultures grown in the Nutrient Broth rich medium (Himedia, India) were inoculated (1 mL) into 150 mL of modified K+-free Hartman’s-de Bont (mHdeB) medium containing (per liter): 11.8 g Na_2_HPO_4_·12H_2_O, 1.7 g citric acid, 20 g (NH_4_)_2_SO_4_, 30 mL glycerol, 0.05% Tween 80, and 10 mL of trace elements solution (1 g EDTA, 10 g MgCl_2_·6H_2_O, 0.1 g CaCl_2_·2H_2_O, 0.04 g CoCl_2_·6H_2_O, 0.1 g MnCl_2_·2H_2_O, 0.02 g Na_2_MoO_4_·2H_2_O, 0.2 g ZnSO_4_·7H_2_O, 0.02 g CuSO_4_·5H_2_O, and 0.5 g FeSO_4_·7H_2_O per liter). The components were mixed, the medium pH adjusted to 7.0, and 0.05% (*v*/*v*) Tween 80 and 0.5% BSA (Cohn-Analog, Sigma, St Louis, MO, USA) were added; for plasmid-containing strains, growth media were supplemented with kanamycin (50 µg/mL). Cultures were incubated at 37 °C on an orbital shaker (200 rpm.)

### 4.11. Viability Assay 

Bacterial suspensions were serially diluted in fresh medium, and 100 μL from each dilution was spread on agar-solidified NBE and incubated at 37 °C. The number of colony-forming units (CFUs) was determined after 5 days.

### 4.12. Resuscitation Assay 

Resuscitation and calculation of most probable numbers (MPNs) of viable *M. smegmatis* cells were performed in 48-well plastic plates (Corning) containing 450 µL Sauton’s medium supplemented with 10^5^−10^6^ *M. luteus* cells from exponentially growing cultures. *M. luteus* cells did not interfere with the calculation of *M. smegmatis* growth as they do not multiply in Sauton’s medium. Ten-fold serially diluted samples of *M. smegmatis* cultures (50 µL) were added to each well and plates were incubated at 37 °C with agitation at 100 rpm for 7 days. Wells with visible bacterial growth were counted as positive and MPN values were calculated using standard statistical methods.

### 4.13. Statistical Analysis

Statistical analysis was performed using Microsoft office Excel 2007 and GraphPad Prism 6.0 (GraphPad Software Inc., La Jolla, CA, USA). The data were expressed as the mean ± SD. For non-normally distributed data, the Mann–Whitney U test was used. Differences were considered statistically significant at *p* < 0.05. At least three independent experiments were performed for each assay.

## Figures and Tables

**Table 1:** Genes differentially expressed in the ΔF6 strain compared to the wt.

Gene	Product	Symbol	Fold Change
*MSMEG_0149*	Thiamine biosynthesis protein (ThiC)		27.3
*MSMEG_0150*	NAD(P) transhydrogenase subunit beta		29.2
*MSMEG_0151*	PntAB protein		39.4
*MSMEG_0152*	Alanine dehydrogenase		24.3
*MSMEG_0153*	2-Dehydropantoate 2-reductase	*panE*	16.3
*MSMEG_0154*	Pyruvate kinase	*pyk*	16.9
*MSMEG_0156*	LysR family transcriptional regulator		14.5
*MSMEG_0157*	Oxalyl-CoA decarboxylase		54.1
*MSMEG_0158*	Formyl-coenzyme A transferase		45.5
*MSMEG_0159*	Formate dehydrogenase subunit gamma		29.8
*MSMEG_0160*	Formate dehydrogenase subunit beta		38.5
*MSMEG_0161*	Formate dehydrogenase subunit alpha		35.6
*MSMEG_0162*	NAD-dependent formate dehydrogenase subunit delta		30.3
*MSMEG_0168*	Formyl-coenzyme A transferase		19.3
*MSMEG_4640*	Hypothetical protein		14.2

## Data Availability

All RNA-seq data generated for this study has been deposited in the GEO repository under accession number GSE149173 (https://www.ncbi.nlm.nih.gov/geo/query/acc.cgi?acc=GSE149173, accessed on 31 May 2021.)

## Supplementary Materials

The following data are available online at https://www.mdpi.com/article/10.3390/ijms222111536/s1.
