# Supplementary material for: Small RNA F6 Provides Mycobacterium smegmatis Entry into Dormancy
Source: Int J Mol Sci. 2021 Oct 26;22(21):11536. doi: 10.3390/ijms222111536 (PMC8583896; doi:10.3390/ijms222111536)
Supplement: Supplementary file 1 [file ijms-22-11536-s001.zip › Supplementary Figure S2.pdf]

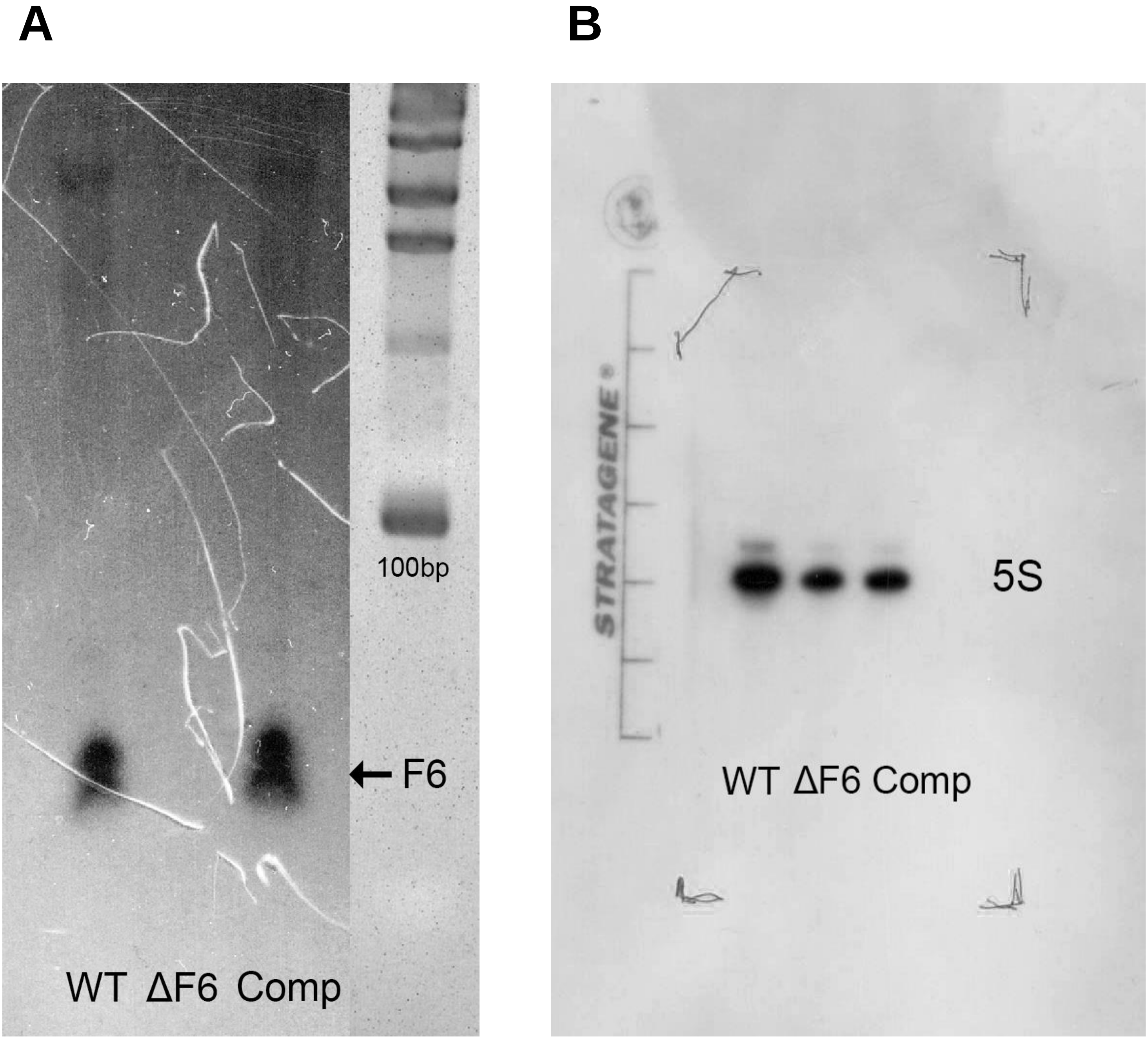

Northern blot analysis of F6 transcription in wt, ΔF6, and F6 complemented (ΔF6:F6) strains (A) and the 5S loading control (B)

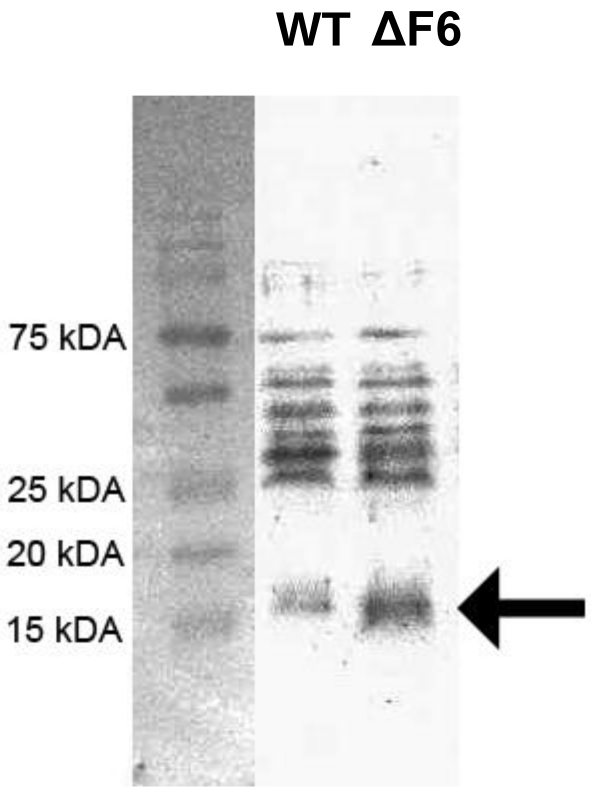

Inhibition of RpfE2 protein synthesis by F6. Wt and ΔF6 cultures were analyzed for RpfE2 protein expression by western blotting using antibodies for Rpf conservative domain.

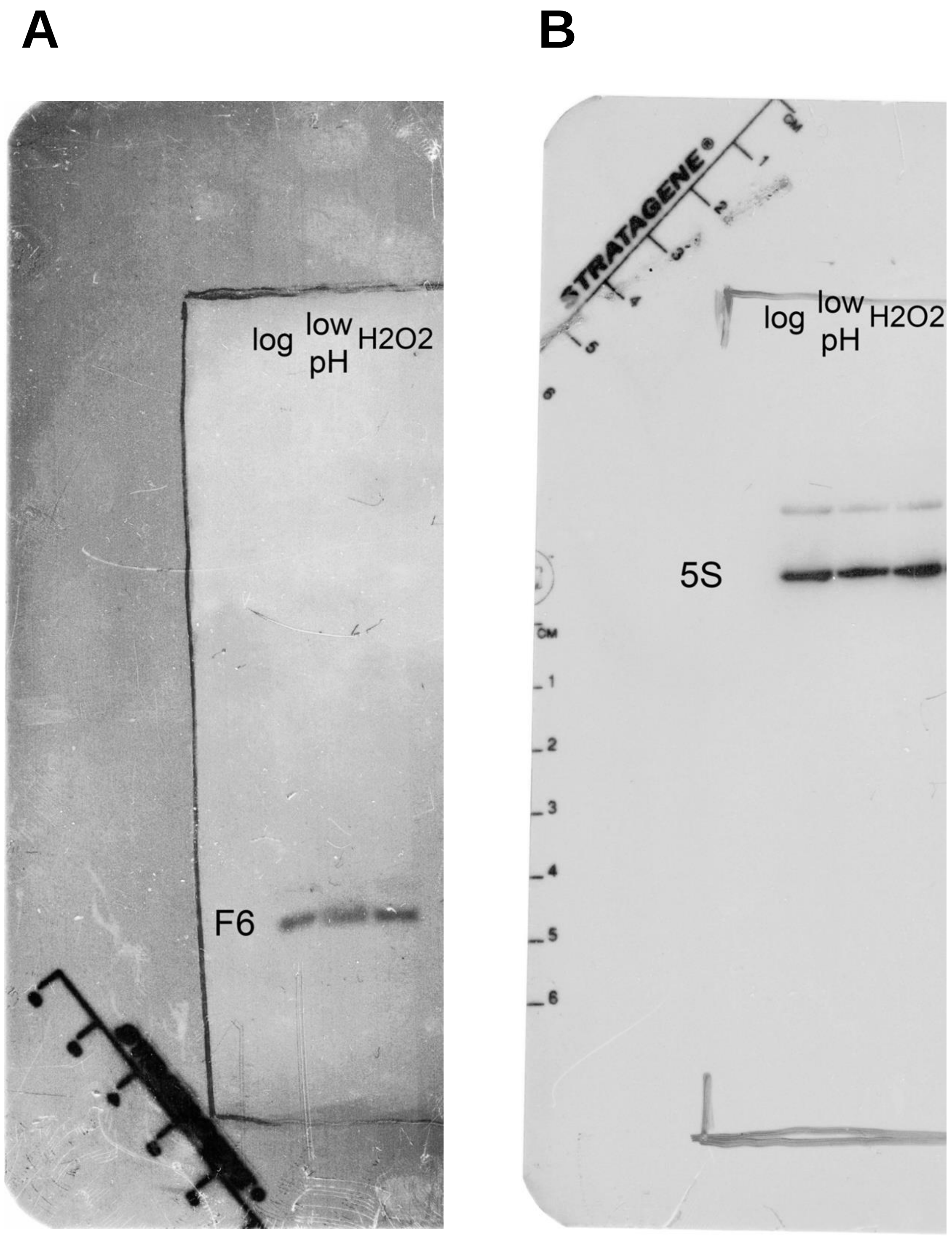

Transcription of F6 in mid-log phase of growth and under acidic (low pH) and oxidative (H2O2) stresses estimated by Northern blotting. (A) and the 5S loading control (B)
