# Supplementary material for: Small RNA F6 Provides Mycobacterium smegmatis Entry into Dormancy
Source: Int J Mol Sci. 2021 Oct 26;22(21):11536. doi: 10.3390/ijms222111536 (PMC8583896; doi:10.3390/ijms222111536)
Supplement: Supplementary file 1 [file ijms-22-11536-s001.zip › Supplementary Table S2.pdf]

Supplementary Table S2.

Oligonucleotides used in the study

Name	Sequence, 5’-3’
Oligonucleotides used for F6 deletion mutant construction	
LHA_F6_R	TCTCTCGAATTCACGCCTCCGTTACACGTCCCGAAAA
RHA_F6_F	TCTCTCGAATTCGGTTCGGGAGTGGCTTGATCCAAAGA
RHA_F6_R	CTCTCTGGATCCCGTGAGGCCGCCGACACCAT
LHA-F6_F	TCTCTCAAGCTTGCCTTTCGCTCGCGGTACTACCT
RHA_F6_seq_R	ATCCCGGAGATGCAGGAGGCCA
RHA_F6_seq_F	CCTGATCAGCGGCGCACACAGATCTT
LHA_F6_seq_F	GCATCGCCGCGGGTCGCTA
LHA_F6_seq_R	GAACGCCTCGTGGATCTCGTAGAAGTCG
F6-KO-check_for	TCGCGGAGAAGAAGAAATCCA
F6-KO-check_rev	TGGGGGCGTCACTACTCGT
Oligonucleotides used for Northern blotting	
NB_F6	GTCGGGTCGGGGGGTCAGACGGCAACACGGAGCTA
NB-5S	GTGGTATCCAACGCAGAGT
Oligonucleotides used for quantitative RT-PCR	
Msm0149-for	ACACCGAAACCGACACCCC
Msm0149-rev	TTGGGGCGCAGACTTTCCC
Msm0150-for	ATCTCTCTGCTGAACGCCTTGAC
Msm0150-rev	AACACGATGGCCGGAATGGA
Msm0157-rev	TTGGTGGTGGCATTGGCAAGAG
Msm0157-for	TTGCGCTACATCGGATTCCGG
Msm0162-for	GCGCACTTCGGTTACCTGC
Msm0162-rev	CGGCGTAGATCAGGCTGAACA

msm4640-for1	GTCAACTGGGACGCCATC
msm4640-rev1	ACGTGGACTGCTTGAACTGC
16S-for	ATGTCGGTTCCCTTGTGGC
16S-rev	CAAGGGTTGCGCTCGTTG
Oligonucleotides used for constructions of plasmids	
UTR4640-for	ACCTCTAGATAGTGATCGCGCGGTTG
UTR4640-rev	CCAGGATCCTGCCGTACAAACGTTC
UTR4640-mut-rev	GACTTCCGACACGAGGACCG
UTR4640_mut-for	TGTGAAGGGCCACGATGATGATG
F6-mut-rev	TGTGGGTCAGACGGCAACACG
F6-mut-for	GACTCGACGGCAACACGGG
4640-for_BamH1	GGATCCGTGAAGAGGGCCTTCG
4640-rev_EcoR1	GAATTCCTAGCGACCGAAGGCG
pAMYC-For	AGCAAGAGATTACGCGCAGAC
pAMYC-Rev	GACAGTCATAAGTGCGGCGA
pMV306-For	TATGGAAAAACGCCAGCAACGC
pMV306-Rev	ATGCCTGGCAGTCGATCGTA

Plasmids used and generated in this study

Plasmid	Description	Reference or source
p2NIL	Cloning vector; Km^r	[1]
pGOAL19	Plasmid carrying *hyg*, *lacZ*, and *sacB* genes in a PacI cassette; Amp^r, Hyg^r	[1]
pF6_new_Knockout	Suicide vector to delete F6 in *M. smegmatis*; Km^r, Hyg^r	This study
pMV306hsp	Integrative shuttle vector, Km^r	gift from Brian Robertson & Siouxsie Wiles (Addgene plasmid # 26155 ;

		http://n2t.net/addgene:26155 ; RRID:Addgene_26155)
pMV306_rrnB	pMV306hsp derivative bearing *Msm rrnB* promoter (-79) and *rrnB* terminator, Km^r<br><br>The fragment Msm *rrnB* promoter-*rrnB* terminator was taken from pMV261_*rrnB* [2] and cloned into pMV306hsp at the XbaI/SalI restriction sites	This study
pMV306_F6	pMV306_rrnB derivative expressing F6 under *Msm rrnB* promoter, Km^r<br><br>F6 gene was amplified from the *M. smegmatis* genomic DNA and cloned into pMV306_rrnB at the BamHI/HindIII restriction sites	This study
pMV306_MSMEG4640$_{5'utr}$ GFP	pMV306_rrnB derivative expressing GFP fused with MSMEG4640 5'UTR under *Msm rrnB* promoter, Km^r<br><br>GFP (a kind gift of Dr. Irina Boni, Shemyakin-Ovchinnikov Institute of Bioorganic Chemistry, Russia) was cloned into pMV306_rrnB at the BamHI/HindIII restriction sites. 5'UTR MSMEG-4640 was amplified with UTR-4640-for/UTR-4640-rev primers and cloned upstream GFP at XbaI/BamHI restriction sites.	This study
pMV306_MSMEG4640$_{5'utr}$ mut_GFP	pMV306_rrnB derivative expressing GFP fused with mutant MSMEG4640 5'UTR, Km^r<br><br>Mutations in 5'UTR4640 were introduced into pMV306_MSMEG4640$_{5'utr}$GFP using a Q5 Site-Directed Mutagenesis Kit (NEB) and primers UTR4640-mut-for/ UTR4640_mut-rev	This study

pAMYC	Replicative shuttle vector, Tet^{r}, Cm^{r} (pACYC184 derivative carrying *oriM*)	[3]
pAMYC_rrnB	pAMYC bearing *Msm rrnB* promoter and *rrnB* terminator, Tet^{r}, Cm^{r}	This study
pAMYC_F6	pAMYC_rrnB derivative expressing F6 under *Msm rrnB* promoter, Tet^{r}, Cm^{r}	This study
pAMYC_F6mut	pAMYC_rrnB derivative expressing mutant F6 gene under *Msm rrnB* promoter, Tet^{r}, Cm^{r}<br>Mutations in the F6 seed were introduced into pAMYC_F6 using a Q5 Site-Directed Mutagenesis Kit (NEB) and primers F6-mut-for/F6-mut-rev	This study

Mycobacterium smegmatis **strains generated in this study**

Strain	Description
ΔF6	*M. smegmatis* mc^{2}155 lacking F6 gene
ΔF6:pMV306	*M. smegmatis* ΔF6 with integrated plasmid pMV306_rrnB (Km^{r})
ΔF6:F6	*M. smegmatis* ΔF6 with integrated plasmid pMV306_F6 (Km^{r})
Msm_GFP	*M. smegmatis* bearing *Msm rrnB'*-*'gfp* reporter in the chromosome (Km^{r})
Msm_MSMEG4640$_{5'utr}$::GFP	*M. smegmatis* bearing *Msm rrnB'*-'MSMEG4640$_{5'utr}$*'*-*'gfp* reporter in the chromosome (Km^{r})
Msm_ MSMEG4640$_{5'utr}$mut::GFP	*M. smegmatis* bearing *Msm rrnB'*-'MSMEG4640$_{5'utr}$mut*'*-*'gfp* reporter
MsmΔ*F6*_MSMEG4640$_{5'utr}$::GFP	*M. smegmatis* Δ*F6* bearing *Msm rrnB'*-'MSMEG4640$_{5'utr}$*'*-*'gfp* reporter
MsmΔ*F6*__*F6*_MSMEG4640$_{5'utr}$::GFP	*M. smegmatis* Δ*F6* bearing F6 gene in the replicative shuttle vector (Cm^{r}) and *Msm rrnB'*-'MSMEG4640$_{5'utr}$*'*-*'gfp* reporter in the chromosome (Km^{r})
MsmΔ*F6*__*F6*mut _MSMEG4640$_{5'utr}$::GFP	*M. smegmatis* Δ*F6* bearing mutant F6 gene in the replicative shuttle vector (Cm^{r}) and *Msm rrnB'*-*'MSMEG4640*$_{5'utr}$*'-gfp* reporter in the chromosome (Km^{r})
MsmΔ*F6*__*F6*mut_MSMEG_4640$_{5'utr}$ mut:: GFP	*M. smegmatis* Δ*F6* bearing mutant F6 gene and *Msm rrnB'*- 'MSMEG4640$_{5'utr}$mut*'-gfp* reporter (Km^{r})

Amp^r, ampicillin resistant; Km^r, kanamycin resistant, Tet^r, tetracycline resistant; Cm^r, chloramphenicol resistant

References:

1. Parish, T.; Stoker, N.G. Use of a flexible cassette method to generate a double unmarked Mycobacterium tuberculosis tlyA plcABC mutant by gene replacement. *Microbiology* **2000**, *146*, 1969-1975.
2. Ignatov, D.V.; Salina, E.G.; Fursov, M.V.; Skvortsov, T.A.; Azhikina, T.L.; Kaprelyants, A.S. Dormant non-culturable Mycobacterium tuberculosis retains stable low-abundant mRNA. *BMC genomics* **2015**, *16*, 1-13.
3. Aseev, L.V., Koledinskaya, L.S., Bychenko, O.S. and Boni, I.V., Regulation of Ribosomal Protein Synthesis in Mycobacteria: The Autogenous Control of rpsO. *International Journal of Molecular Sciences*, **2021**. *22*(18), p.9679.
