# Supplementary figures and images for: Small RNA F6 Provides Mycobacterium smegmatis Entry into Dormancy
Source: Int J Mol Sci. 2021 Oct 26;22(21):11536. doi: 10.3390/ijms222111536 (PMC8583896; doi:10.3390/ijms222111536)

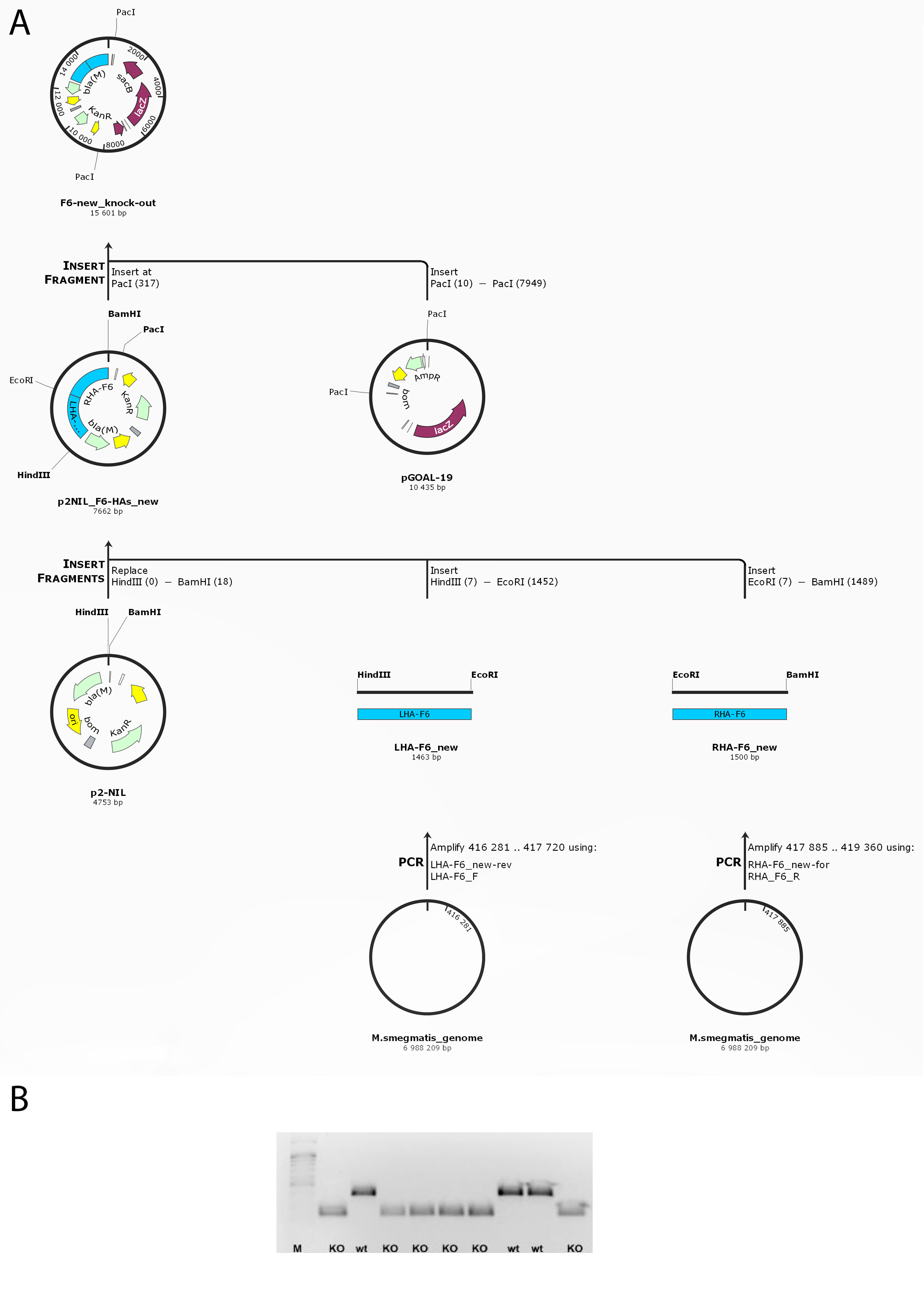

Supplement: Supplementary file 1 [file ijms-22-11536-s001.zip › Supplementary Figure S1.tif]
